# Hot spots and frontiers of postpartum depression research in the past 5 years: A bibliometric analysis

**DOI:** 10.3389/fpsyg.2022.901668

**Published:** 2022-12-20

**Authors:** Runfang Wang, Yaokun Shan

**Affiliations:** Medical School of Rehabilitation, Shandong University of Traditional Chinese Medicine, Jinan, Shandong, China

**Keywords:** postpartum depression, CiteSpace, bibliometric, review, visualization

## Abstract

**Objective:**

This study applied the bibliometric method to analyze the hotspots and frontiers in the field of postpartum depression research from 2017 to 2021.

**Methods:**

A computer-based search for studies related to postpartum depression (PPD) was conducted in the Web of Science Core Collection from 2017 to 2021. Using CiteSpace 5.8. R3 visualization software, this paper analyzed the hot spots and frontiers of postpartum depression research from countries, institutions, authors, cited references and keywords, and drew corresponding knowledge maps.

**Results:**

A total of 3,789 articles were retrieved, and the annual publication volume showed a steady increase. The countries and institutions with the most publications were the United States and the University of Toronto, respectively. Cindylee Dennis was the most productive author. The most frequently cited and centrally cited articles were meta-analyses on risk factors associated with antenatal depression or anxiety and meta-analyses on the prevalence of antenatal or postnatal anxiety, respectively. The research hotspots in the field of postpartum depression in the past 5 years mainly focused on the risk factors for PPD, and the impact of the COVID-19 epidemic on PPD. Providing various aspects of social and family support to women with PPD may be a research trend in this field.

**Conclusion:**

This study provides a trend and frontier in the field of postpartum depression, and valuable information for researchers to find potential partners and partner countries, and a reference for future research topics and development directions.

## Introduction

Postpartum depression (PPD) is a serious clinical disease, and the typical symptoms mainly include anxiety, irritability, anxiety, loss of appetite, sleep disorders, etc. PPD has a significant impact on the emotional, personality and cognitive development of mothers, partners, families and infants (Sun et al., 2021; Walker et al., 2021). The pathological mechanism of PPD is relatively complex. Current studies have proved that both Estrogen withdrawal and prolactin level regulation may affect the occurrence of PPD (Li et al., 2018; Wang et al., 2020; Cheng et al., 2022). According to statistics, nearly 15% of women worldwide suffer from PPD (Figueiredo et al., 2021). Compared with men, women are twice as likely to suffer from depression (Martini et al., 2009). Changes in hormone levels in women during puberty, pregnancy and menopause may be an important cause of depression in women (Martini et al., 2009). Especially after delivery, poor maternal physical condition, poor quality of life and other risk factors that may lead to depression increase the likelihood of PPD (Julian et al., 2021; Walker et al., 2021). Research has shown that more than 20% of women with PPD have bipolar disorder (Wisner et al., 2013). Gollan et al. (2021) found that anhedonic symptoms were more common in women with postpartum bipolar disorder than in those with unipolar depression and twice as high. Therefore, the screening of PPD is very important. The Edinburgh Postnatal Depression Scale (EPDS) is the most widely used screening tool. Screening women with possible PPD using scales and clinical symptoms can help differentiate bipolar disorder from PPD and avoid delays in treatment (Clark et al., 2022).

CiteSpace was created by Professor Chen Chaomei of Drexel University in the United States. It is a knowledge management software for bibliometric analysis (Shou et al., 2022). By generating a visual knowledge map, CiteSpace explores the research status, research hotspots, research fronts and evolution process of a scientific field, reveals research directions, research stages and frontier characteristics, and finally judges the development trend of the field (Luo et al., 2021). So far, CiteSpace software has been used in various fields such as environmental science, education and public health (Dang et al., 2021). This paper used CiteSpace 5.8. R3 software to analyze the characteristics of PPD research literature listed in the Web of Science database in the past 5 years. Focusing on high-impact countries, institutions, authors, cited literature, high-frequency keywords and keywords with citation bursts, the hotspots and frontier trends of PPD research were analyzed and tracked to provide some reference for the research in this field and the development of related disciplines.

## Data and methods

### Data and retrieval strategy

The Science Citation Index Expanded (SCI-EXPANDED) database in the Web of Science Core Collection was searched by computer using the search strategy: TS = (“postpartum depression” OR “postpartum depression therap*”). The time span was set to 2017–2021. The last retrieval date was 2022-07-12. The document type was limited to “Article,” and the language type was set to “English.”

### Methods

The full records of the retrieved data and references were downloaded in plain text format and named “download_XXX.” CiteSpace 5.8. R3 visualization software was used to visualize the information from the literature search according to the first author in the article as well as countries, authors, keywords, research institutions and so on. In the parameter setting for the CiteSpace 5.8. R3 software, the time span was set to 2017–2021, the time scale was set to 1 year, and the threshold term was selected as “TopN,” which was set to 50. The “Pathfinder” and “Pruning Sliced Networks” options were selected as the shear connections to simplify the network structure and highlight important features. For node types, countries, institutions, authors, references and keywords were selected for co-occurrence analysis, and visual maps were drawn(Chen, 2004). The use of CiteSpace software highlights the word detection function to explore the keyword surge rate of change, and generate a high-intensity mutation rate of the keyword ranking table to achieve an analysis of the status quo and predict future development (Zhu et al., 2021). Nodes and links were used to generate visual knowledge maps. Each node in the figure represents an element to be analyzed, such as reference literature, country or institution. The size of the nodes represents the frequency of reference, and nodes with different colors represent different years. The connection lines between nodes represent the co-occurrence or co-citation relationship, the thickness of the line represents the strength of the relationship, and the corresponding color of the node represents the first co-occurrence or co-citation time. The purple outer ring of the node represents centrality. The thicker the purple outer ring is, the higher the centrality is. Nodes with high centrality (> 0.1) are usually considered research hotspots or turning points in this field (Luo et al., 2021; Zhong et al., 2022).

## Results

A total of 3,871 articles were retrieved. The included literatures were screened using CiteSpace to exclude duplicate literatures. To ensure that the topics and main content of the literature were related to postpartum depression, the literature was reread, and the abstracts were screened to exclude irrelevant literature. A total of 3,789 papers were included after excluding conference abstracts, letters, reviews, and periodical news reviews.

### Publication outputs

**Figure 1:**
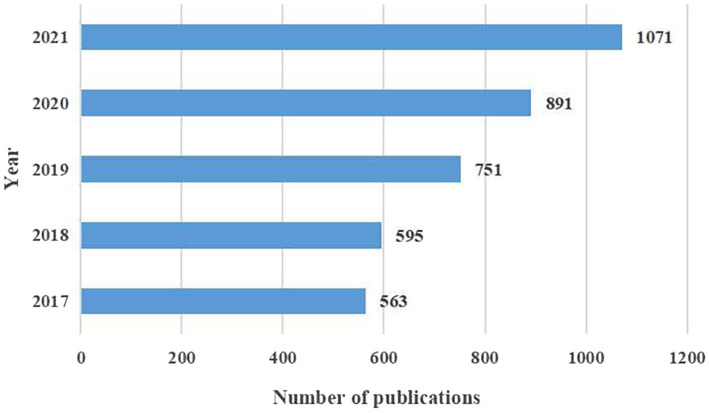
The annual number of publications.

The average annual published articles of the PPD research field from 2017 to 2021 was 757.8, showing a gradual increasing trend, with the largest increase in 2020–2021. The number of publications in 2021 was 1.9 times that in 2017. This shows that an increasing number of researchers have begun to pay attention to the study of PPD (Figure 1).

### Countries analysis

**Figure 2:**
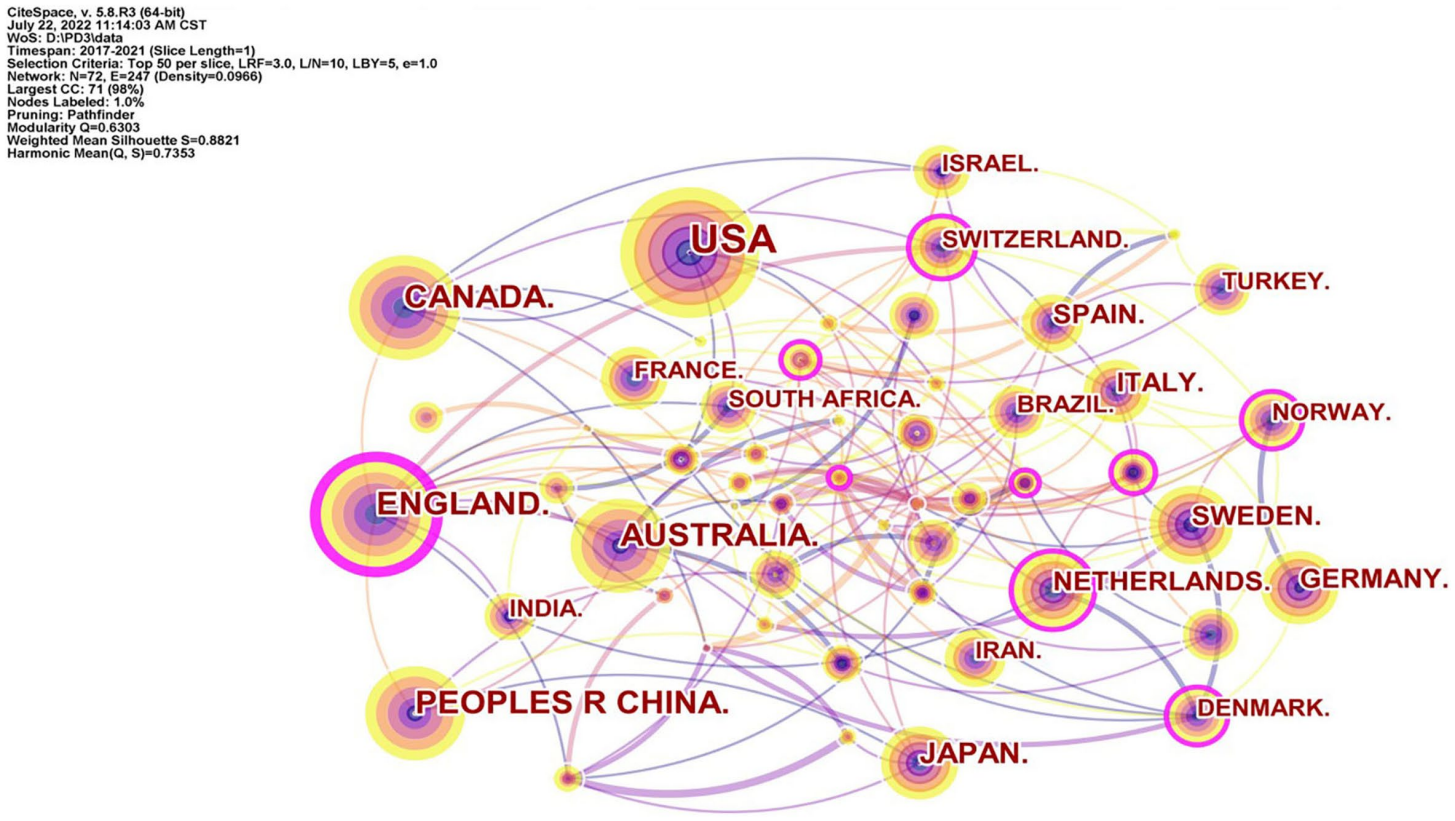
The cooperation network map of countries.

The cooperation network map of countries is shown in Figure 2. The country with the largest number of articles from 2017 to 2021 was the United States, with 1,300 articles. The country with the second most articles was Canada, with 378 articles. The country with the third most articles was England, with 373 articles. Ten countries had > 100 articles. The centrality of England, 0.23, was the highest, 0.23, followed by Croatia (0.17) and Greece and Malawi (both 0.14; Table 1). High centrality indicates that these countries have a strong influence and high position in PPD research.

**Table 1:** Top 10 productive countries in the field of postpartum depression (PPD).

Rank	Count of articles	Country	Centrality
1	1,300	United States	0.03
2	378	Canada	0.09
3	373	England	0.23
4	339	China	0.03
5	289	Australia	0.07
6	151	Japan	0.04
7	139	Germany	0.07
8	137	Netherlands	0.13
9	119	Sweden	0.06
10	112	Italy	0.05

### Institutions analysis

**Figure 3:**
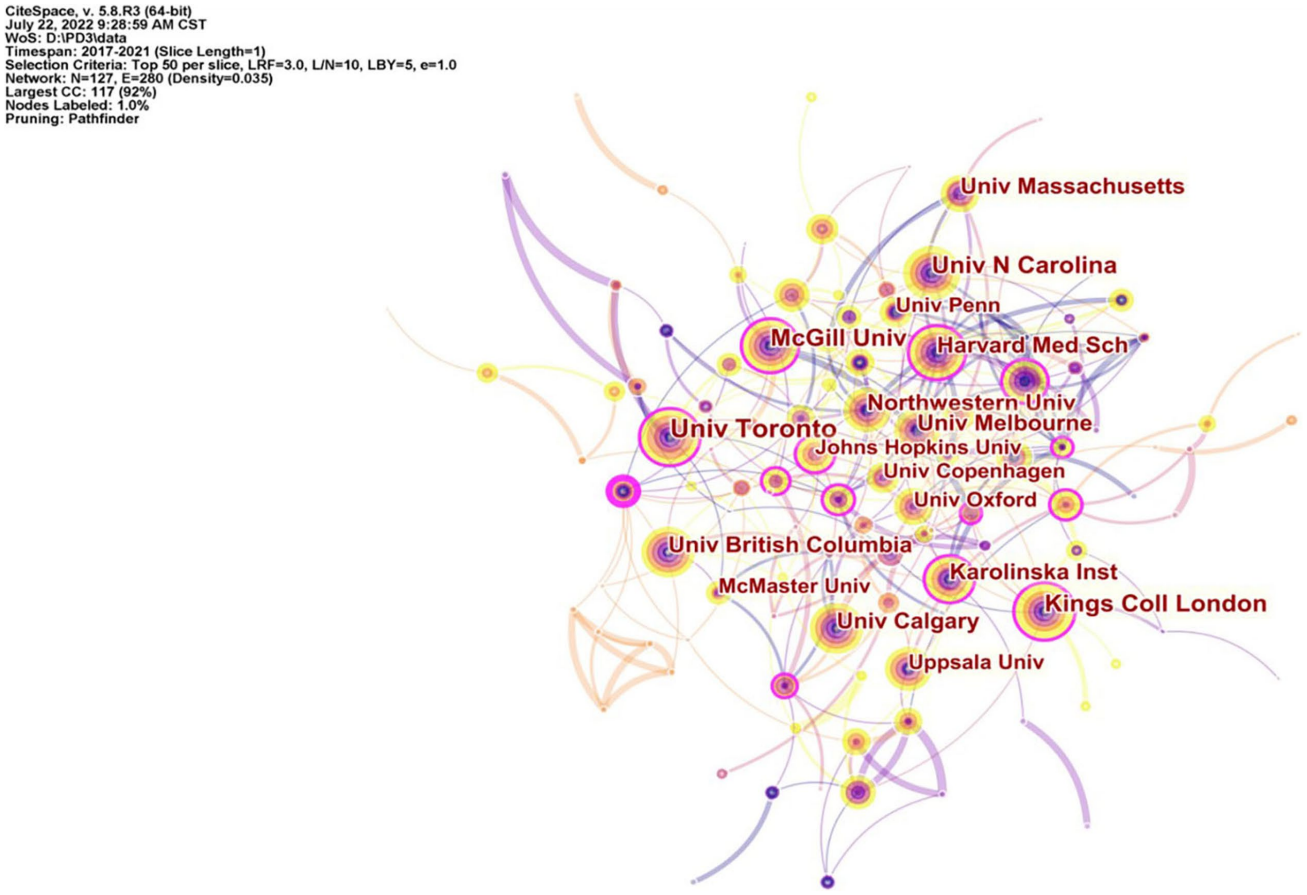
The cooperation network map of institutions.

Figure 3 is the cooperation network map of institutions. The most published was the University of Toronto in Canada, with 92 articles. The University of North Carolina in the United States and King’s College London in the United Kingdom were ranked second, each with 85 articles published. The third most published institution was McGill University in Canada (Table 2). The visualization of research institutions showed that the top three institutions with centrality were McGill University (0.21), University of Melbourne (0.16) and King’s College London (0.13), which had established good cooperation with other institutions (Figure 3).

**Table 2:** Top 10 productive institutions in the field of PPD.

Rank	Count of articles	Institution	Centrality
1	92	University of Toronto	0.17
2	85	University of North Carolina	0.08
3	85	King’s College London	0.17
4	69	McGill University	0.11
5	61	University of Calgary	0.01
6	60	University of British Columbia	0.01
7	59	Northwest University	0.05
8	58	University of Melbourne	0.06
9	58	Harvard Medical School	0.19
10	53	University of Massachusetts	0.04

### Author analysis

**Figure 4:**
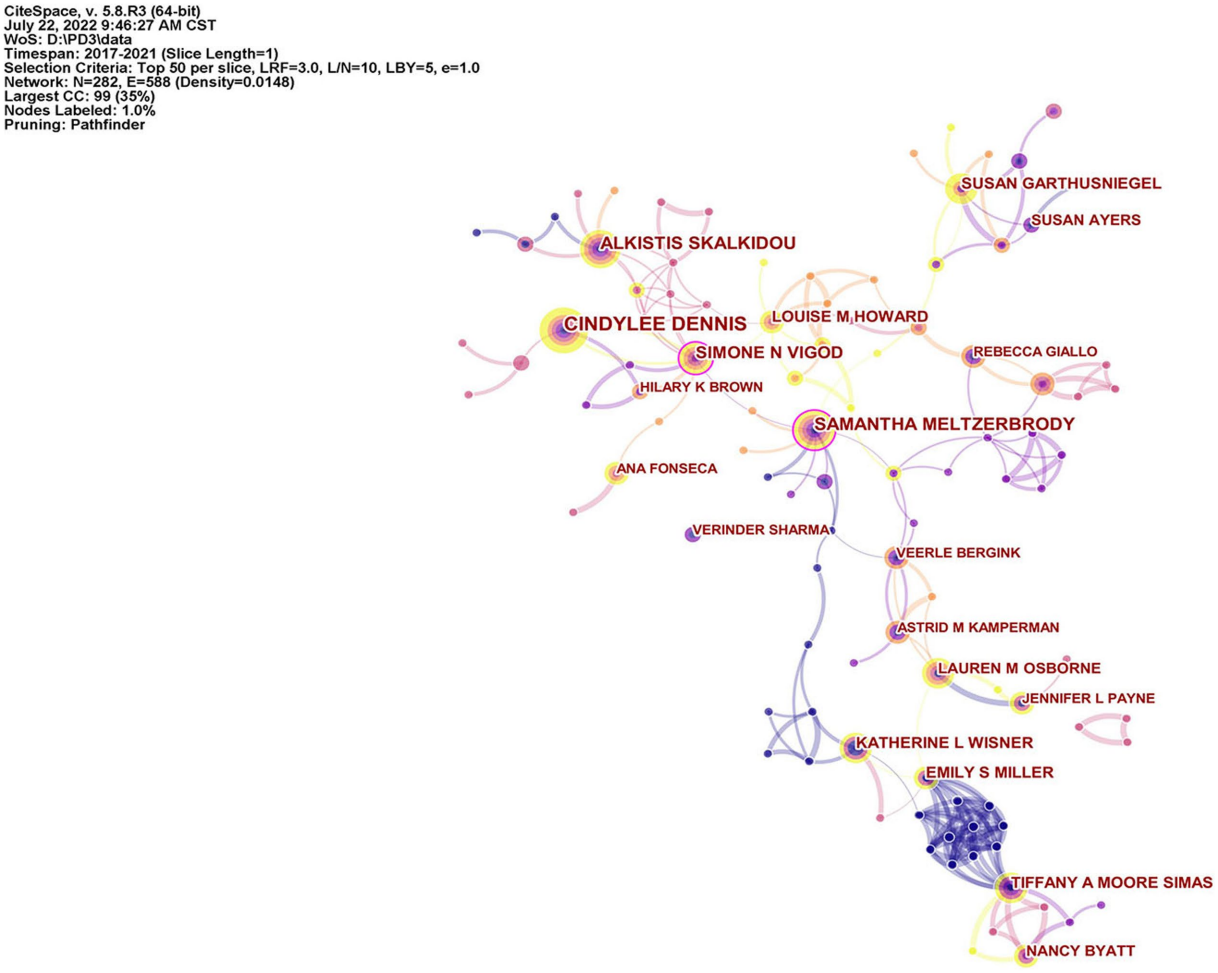
The visualization map of authors.

The visualization map of authors is shown in Figure 4. Among them, 29 authors published more than 10 articles. Cindylee Dennis, Samantha Meltzer-Brody and Alkistis Skalkidou published 34, 27 and 26 articles, respectively. The author with the highest centrality was Samantha Meltzer-Brody, with centrality of 0.13; next were Simone N Vigod and Lauren M Osborne, with centrality of 0.1 and 0.09, respectively (Table 3). The analysis results show that Professor Dennis had the highest number of published papers in this field in the past 5 years, with more research results, while Professor Meltzer-Brody had a greater influence on the research. There was a certain degree of cooperation among the author teams, and the author team with the highest number of published papers cooperated more closely.

**Table 3:** TOP 10 productive authors in the field of PPD.

Rank	Count of articles	Author	Centrality
1	34	Cindylee Dennis	0.01
2	27	Samantha Meltzerbrody	0.13
3	26	Alkistis Skalkidou	0.02
4	21	Simone N Vigod	0.1
5	18	Louise M Howard	0.02
6	18	Katherine L Wisner	0.01
7	17	Susan Garthusniegel	0.02
8	17	Megan Galbally	0
9	16	Tiffany A Moore Simas	0.03
10	15	Emily S Miller	0.08

### Cited references analysis

#### Important references

**Figure 5:**
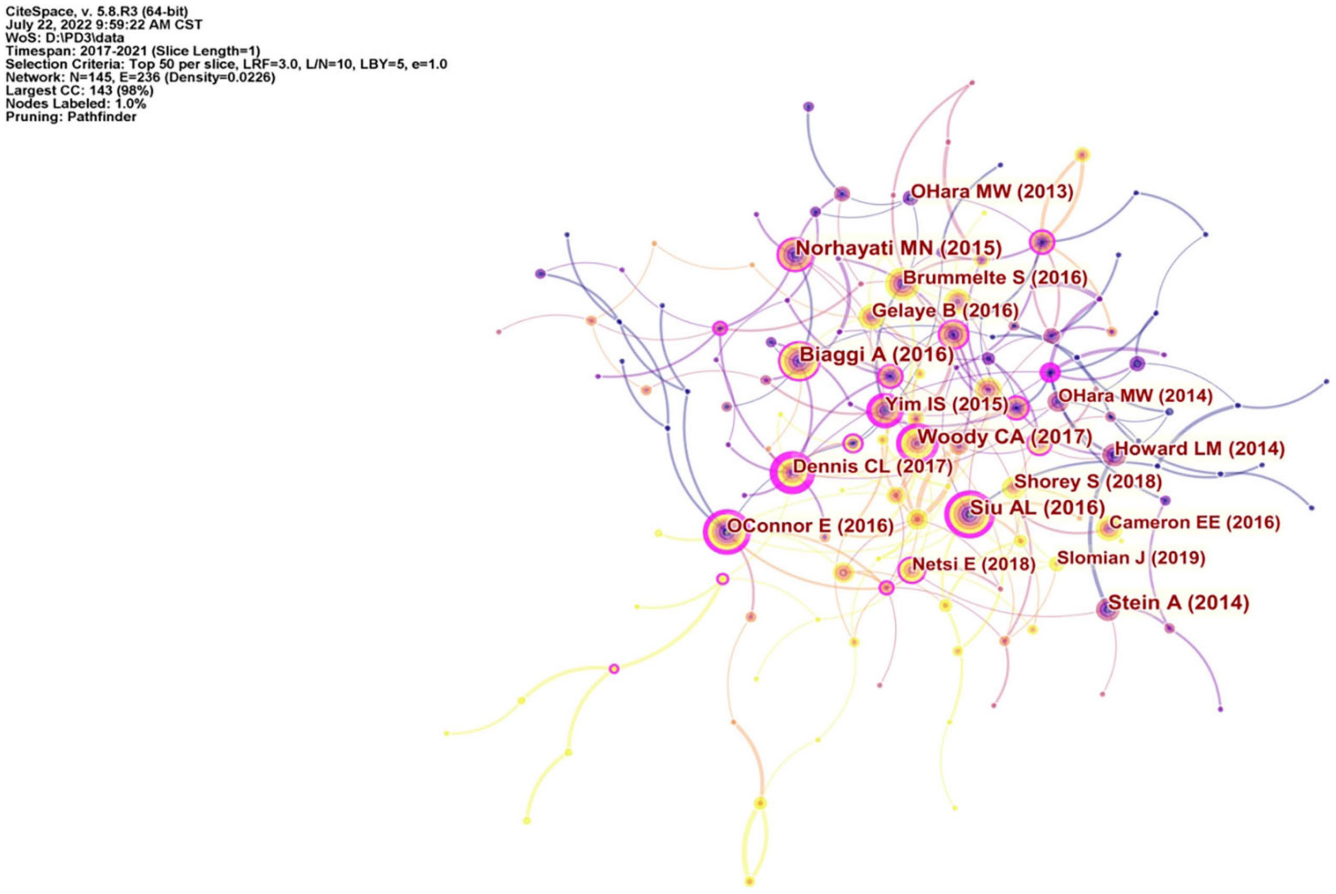
The network map of the co-cited references.

The network map of the co-citation reference analysis was composed of 145 nodes and 236 links (Figure 5). Table 4 shows the top 10 literature information of citation frequency in the co-cited literature. The articles with the highest citation frequency came from the *Journal of Affective Disorders*, with 175 citations. Biaggi et al. (2016) conducted a meta-analysis of relevant literature in PubMed and other databases and found that the most relevant factors for prenatal depression or anxiety included lack of partner or social support, history of abuse or domestic violence, and history of personal psychosis. Understanding the risk factors was helpful to screen women with anxiety and depression risks during pregnancy to prevent the transition from prenatal depression to postpartum depression. The articles with the highest centrality came from *The British Journal of Psychiatry*, with a centrality of 0.50. Dennis et al. (2017a) conducted a meta-analysis of the studies published before January 2016 on the prevalence of prenatal or postnatal anxiety and found that the incidence of perinatal anxiety was high, which is worthy of clinical attention.

**Table 4:** Top 10 co-cited references ranked by the number of citations.

Rank	First author	Country	Year	Count	Centrality	Article type	Number of included studies	Results reported in the article
1	Biaggi A	United Kingdom	2016	175	0.11	Systematic review	97 studies	The administration of a screening tool to identify women at risk of anxiety and depression during pregnancy should be universal practice in order to promote the long-term wellbeing of mothers and babies, and the knowledge of specific risk factors may help creating such screening tool targeting women at higher risk.
2	Siu AL	United States	2016	155	0.27	Recommendation statement	Not reported	It was suggested that depression screening should be carried out in the general adult population, including pregnant women and postpartum women.
3	Woody CA	Australia	2017	153	0.25	Systematic review and meta-analysis	101 studies	Perinatal depression appears to impose a higher burden on women in low-and middle-income countries.
4	Stein A	United Kingdom	2014	130	0.06	Systematic review and meta-analysis	56 studies	They emphasized the need to detect high-risk parents early and carry out more early intervention and prevention research, especially in socio-economic vulnerable groups and low-income countries.
5	Norhayati MN	Malaysia	2015	129	0.14	Systematic review	202 studies	The current prevalence of postpartum depression is much higher than that previously reported, and similar risk factors are documented. A culturally sensitive cut-off score with adequate psychometric properties of the screening instruments should be available. In future studies, examining the physical, biological, and cultural factors in qualitative studies and in those with adequate methodological qualities is recommended.
6	OConnor E	United States	2016	123	0.28	Evidence report and systematic review	478 studies	Direct and indirect evidence suggested that screening pregnant and postpartum women for depression may reduce depressive symptoms in women with depression and reduce the prevalence of depression in a given population. Evidence for pregnant women was sparser but was consistent with the evidence for postpartum women regarding the benefits of screening, the benefits of treatment, and screening instrument accuracy.
7	Gelaye B	United States	2016	118	0.07	Systematic review and meta-analysis	97 studies	Perinatal depression in low-income and middle-income countries is highly prevalent, affecting about one in four women antepartum and one in five women *post partum*. Mental health policy is likely to become part of country development plans and of bilateral and multilateral development assistance. The mental health of mothers during pregnancy and after childbirth ought to be included in this agenda.
8	Dennis CL	Canada	2017	117	0.5	Systematic review and meta-analysis	102 studies	Results suggest perinatal anxiety is highly prevalent and merits clinical attention. Research is warranted to develop evidence-based interventions.
9	Shorey S	Singapore	2018	101	0.07	Systematic review and meta-analysis	58 studies	This review reports a similar prevalence rate of postpartum depression among mothers without history of depression when compared to mothers with history of depression. Thus, future studies should place equal emphasis on this neglected group of mothers so that targeted interventions and follow-ups can be introduced at appropriate time points.
10	Yim IS	United States	2015	99	0.26	Systematic review and call for integration	199 studies	The strongest PPD risk predictors among biological processes are hypothalamic–pituitary–adrenal dysregulation, inflammatory processes, and genetic vulnerabilities. Among psycho-social factors, the strongest predictors are severe life events, some forms of chronic strain, relationship quality, and support from partner and mother. Fully integrated biopsychosocial investigations with large samples are needed to advance our knowledge of PPD etiology.

Through statistical literature analysis, it was found that the top ten cited literatures were review articles, nine of which were reviews or meta-analysis, and one was a statement of recommendations. Therefore, we also counted the top ten original articles cited frequently, as shown in Table 5. Among them, the most frequently cited article was published in *JAMA Psychiatry Journal*, Wisner et al. (2013) screened postpartum women for PPD through the Edinburgh Postnatal Depression Scale (EPDS). A total of 10,000 mothers were screened, 1,396 (14.0%) of whom were positive. The results showed that the most common primary diagnosis was monophasic depressive disorder (68.5%), and nearly two thirds had comorbidity of anxiety disorders. 22.6% suffered from bipolar disorder. Fairbrother et al. (2016) and other articles published on the Journal of Effective Disorders in 2016 were the most central and original research in this field in the past 5 years. This article mainly studied the influence of anxiety and related diseases on postpartum women. The contents of these original studies mainly included the risk factors of PPD, drug treatment, strategies for prevention and screening of PPD, and peripartum annex and destructive disorders.

**Table 5:** Top 10 original research articles cited frequently in PPD field.

Rank	First author	Country	Year	Count	Centrality	Participants			Results reported in the article
						Sample size	Gender	Age range	
1	Wisner KL	United States	2013	65	0.04	10,000	Female	Not reported	The most common diagnosis in screen-positive women was major depressive disorder with comorbid generalized anxiety disorder. Strategies to differentiate women with bipolar from unipolar disorders are needed.
2	Putnam KT	United States	2017	58	0.08	663	Female	19–40 year old	There might be different types and severity of perinatal depression with varying time of onset throughout pregnancy and *post-partum*. These findings support the need for tailored treatments that improve outcomes for women with perinatal depression.
3	Kanes S	United States	2017	51	0.06	21	Female	18–45 year old	In women with severe post-partum depression, infusion of brexanolone resulted in a significant and clinically meaningful reduction in HAM-D total score, compared with placebo. The results support the rationale for targeting synaptic and extrasynaptic GABAA receptors in the development of therapies for patients with post-partum depression.
4	Martini J	Germany	2015	51	0.14	306	Female	18–40 year old	Peripartum time is a sensitive period for a considerable incidence or persistence/ recurrence of anxiety and depressive disorders albeit the course may be rather heterogeneous. Interventional studies are needed to examine whether an alteration of associated factors could help to prevent peri-partum anxiety and depressive disorders.
5	Osborne LM	United States	2017	41	0	60	Female	Not reported	They found that, after adjustment, every additional ng/mL of T2 ALLO resulted in a 63% (95% CI 13 to 84%, *p* = 0.022) reduction in the risk of developing PPD. Their findings extend previous work connecting ALLO and depression within pregnancy, and indicate that the relationship between pregnancy ALLO and PPD is worth further exploration in a larger sample.
6	Fairbrother N	Canada	2016	38	0.19	310	Female	Not reported	This study provides evidence that, as a group, anxiety and related conditions affect a significant proportion of postpartum women, and are more prevalent than is postpartum depression.
7	Figueiredo B	United States	2014	32	0.08	145	Female	Not reported	These findings suggest that screening for depression symptoms during pregnancy can help to identify women at risk for early cessation of exclusive breastfeeding, and that exclusive breastfeeding may help to reduce symptoms of depression from childbirth to 3 months postpartum.
8	Gaillard A	France	2014	31	0.05	312	Female	31 year old (mean age)	Depression during pregnancy, history of physical abuse, migrant status and postpartum physical complications are four major risk factors for postpartum depression.
9	Dubber S	Germany	2015	29	0.01	80	Female	24–44 year old	Early identification of bonding impairment during pregnancy and postpartum depression in mothers plays an important role for the prevention of potential bonding impairment in the early postpartum period.
10	Wu YT	China	2020	29	0	4,124	Female	Not reported	Major life-threatening public health events such as the COVID-19 outbreak may increase the risk for mental illness among pregnant women including thoughts of self-harm. Strategies targeting maternal stress and isolation such as effective risk communication and the provision of psychological first aid may be particularly useful to prevent negative outcomes for women and their fetuses.

#### Co-citation references clustering and timeline diagram

**Figure 6:**
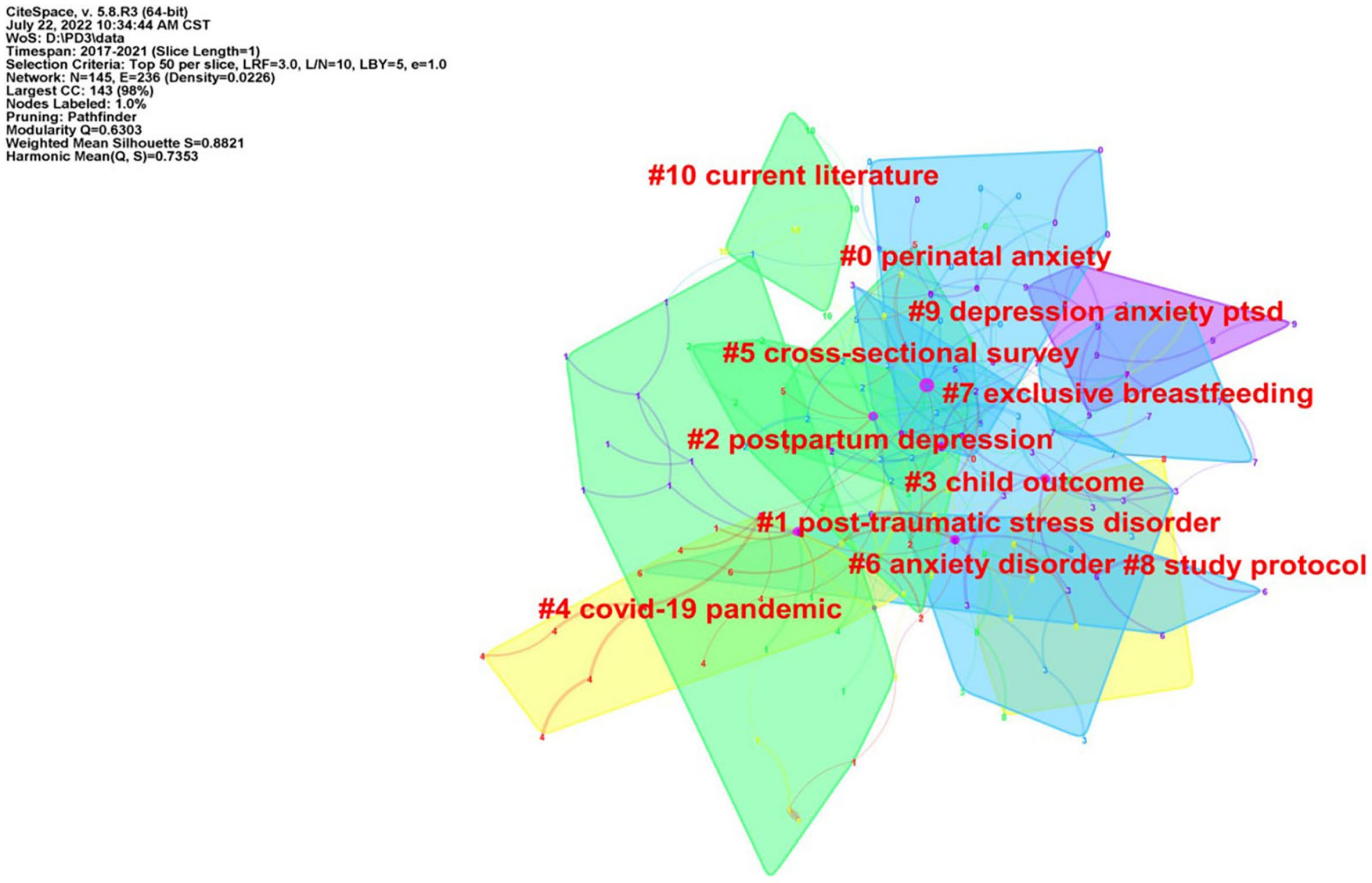
Clustering map of co-cited references.

**Figure 7:**
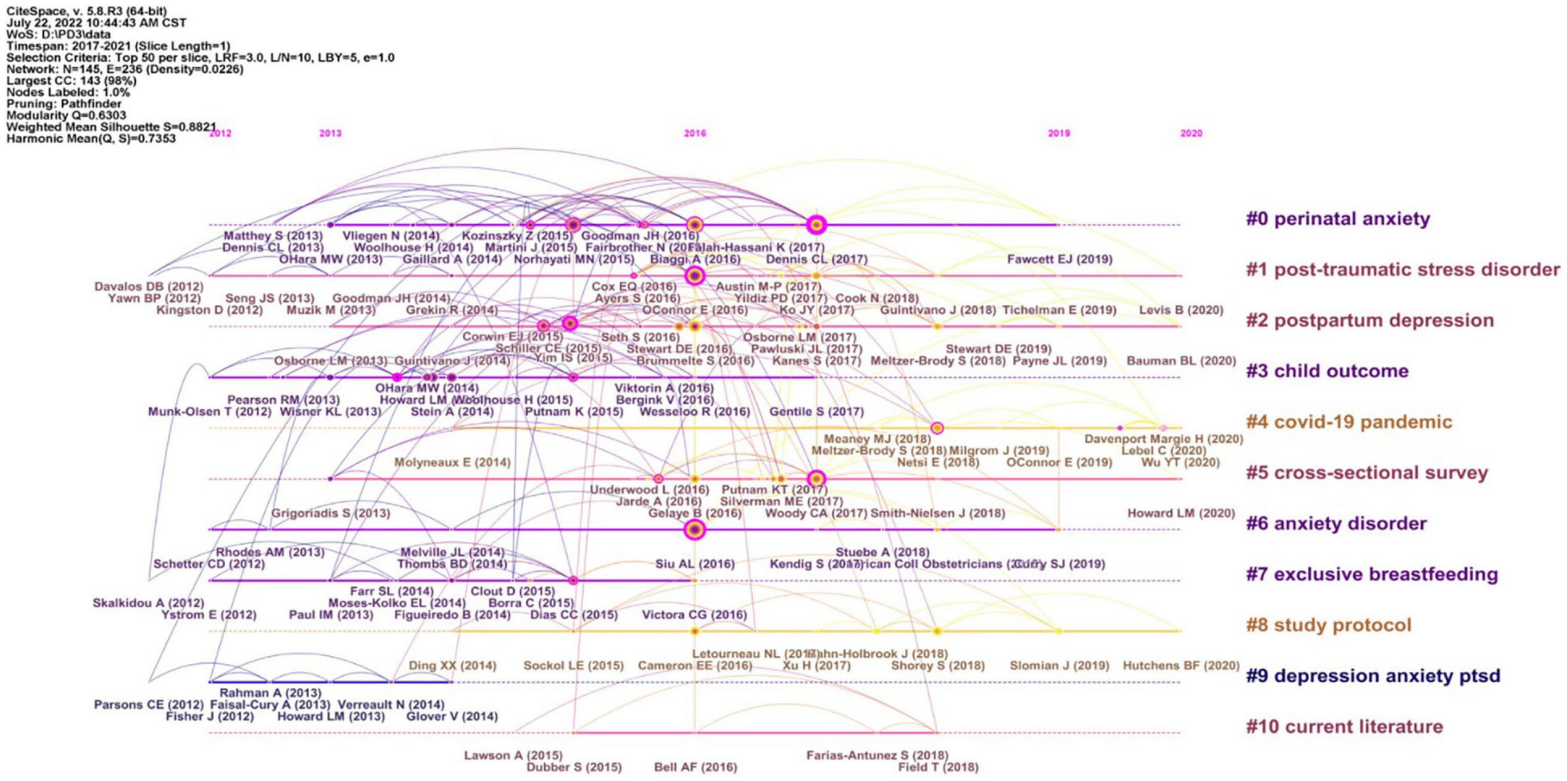
The timeline view of co-cited references.

Cluster analysis was performed on the co-cited documents, and the LLR algorithm was used for clustering to form 11 cluster labels (Figure 6). There were 145 nodes and 236 links in the map, and the silhouette of the map was 0.8821, indicating that the clustering results are meaningful. If the contour value is greater than 0.7, the homogeneity of the clustering members is high, indicating that the clustering results are meaningful. If the value exceeds 0.5, clustering is usually considered reasonable (Guo et al., 2022). The 10 cluster labels were perinatal anxiety (No. 0), post-traumatic stress disorder (No. 1), postpartum depression (No. 2), child outcome (No. 3), COVID-19 pandemic (No. 4), cross-sectional survey (No. 5), anxiety disorder (No. 6), exclusive breastfeeding (No. 7), study protocol (No. 8), depression anxiety PTSD (No. 9) and current literature (No. 10; Table 6). The timeline view of cited references clustering is shown in Figure 7.

**Table 6:** Co-citation cluster information of references in the field of PPD.

Cluster ID	Count	Silhouette	Mean (Year)	Label (LLR)	Contents
0	21	0.953	2015	perinatal anxiety	perinatal anxiety; anxiety symptom; longitudinal cohort study; Chinese–Canadian women; psychometric properties
1	19	0.917	2015	post-traumatic stress disorder	post-traumatic stress disorder; following childbirth; peripartum depression; preterm birth; early recognition
2	18	0.784	2016	postpartum depression	postpartum depression; gaba-a receptor; major depressive disorder; pathophysiological mechanism; maternal reward system
3	18	0.857	2014	child outcome	child outcome; perinatal mental health care; mood disorder; self-reported peripartum; population-based study
4	12	0.782	2018	COVID-19 pandemic	COVID-19 pandemic; seeking treatment; cross-national study; high-risk pregnancy; rapid evidence review
5	12	0.897	2016	cross-sectional survey	cross-sectional survey; south African birth cohort study; placebo-controlled trial; omega-3 fatty acid; postnatal symptom severity
6	12	0.957	2015	anxiety disorder	anxiety disorder; consensus bundle; perinatal depression; maternal mental health; perinatal mood
7	10	0.866	2014	exclusive breastfeeding	exclusive breastfeeding; breastfeeding self-efficacy; maternal satisfaction; mother–child cohort; Crete Greece
8	9	0.923	2017	study protocol	study protocol; postpartum depression; study protocol; controlled trial; prevalence correlate; paternal depression
9	7	0.879	2013	depression anxiety PTSD	depression anxiety PTSD; young adult mother; urban slum; early child development intervention; Karachi Pakistan
10	5	0.889	2016	current literature	current literature; postpartum stress; postpartum anxiety; partnership satisfaction; maternal-fetal attachment

### Keyword analysis

#### Keyword co-occurrence analysis

**Figure 8:**
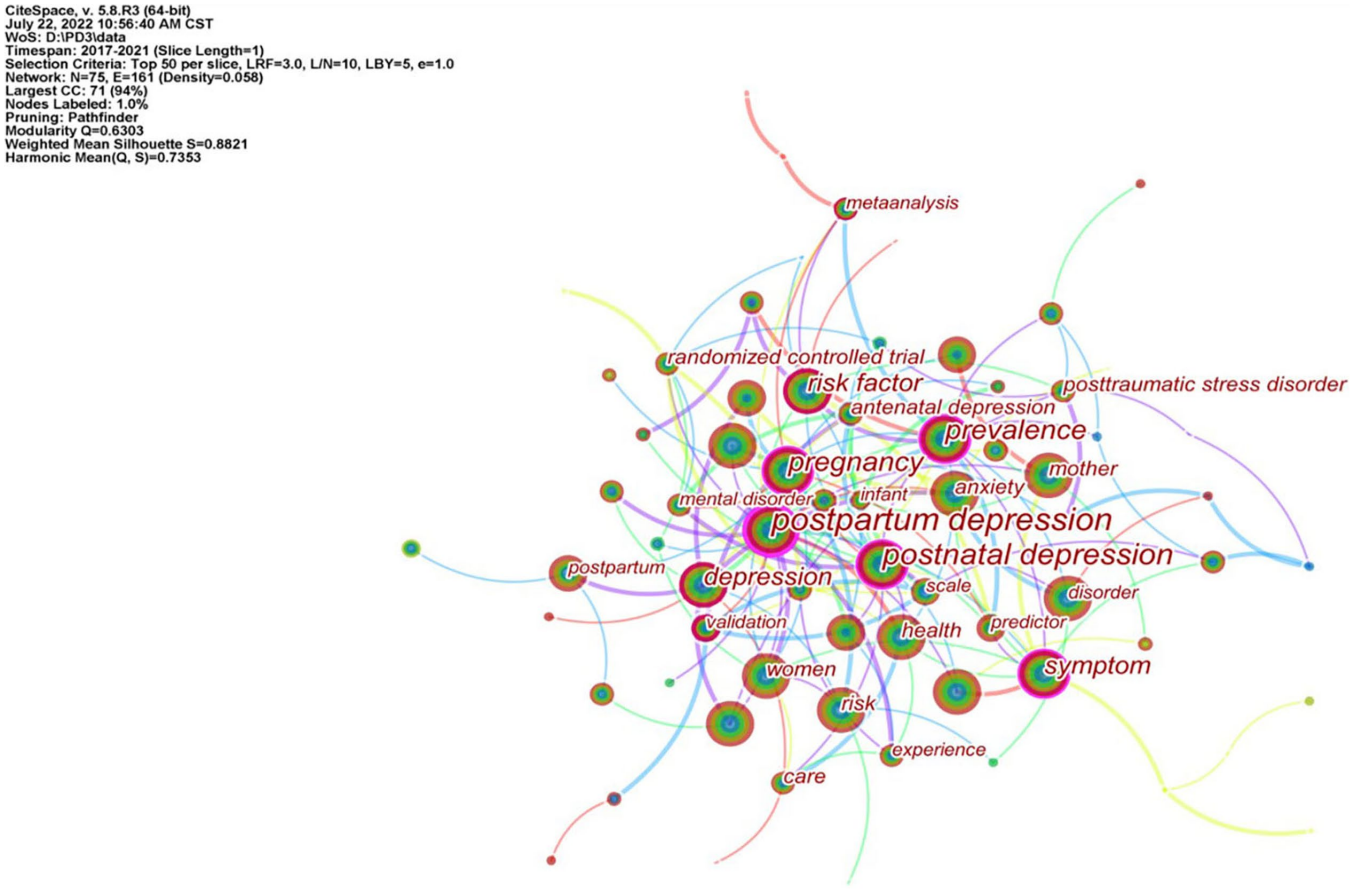
The network map of keywords analysis.

The research hotspots and frontiers in the field of postpartum depression were explored by analyzing the frequency and centrality of keywords. The network map of keyword analysis was composed of 75 nodes and 161 links (Figure 8). Through keyword co-occurrence analysis, the keywords whose frequency and centrality were in the top 10 were statistically ranked, as shown in Table 7. Removing the first search subject term “postpartum depression,” the top 3 keywords with regard to frequency were postnatal depression (1,018), pregnancy (864) and women (830); the top 3 keywords with regard to centrality were pregnancy (0.29), symptoms (0.27) and prevalence (0.26).

**Table 7:** TOP 10 Keywords in terms of frequency and centrality in the field of PPD.

Rank	Keywords	Frequency	Rank	Keywords	Centrality
1	postpartum depression	1,462	1	postpartum depression	0.55
2	postnatal depression	1,018	2	pregnancy	0.29
3	pregnancy	864	3	symptom	0.27
4	women	830	4	prevalence	0.26
5	prevalence	779	5	postnatal depression	0.23
6	symptom	710	6	depression	0.15
7	risk factor	653	7	father	0.15
8	mental health	518	8	risk factor	0.13
9	depression	494	9	validation	0.11
10	mother	465	10	anxiety	0.09

#### Keywords with citation burst

**Figure 9:**
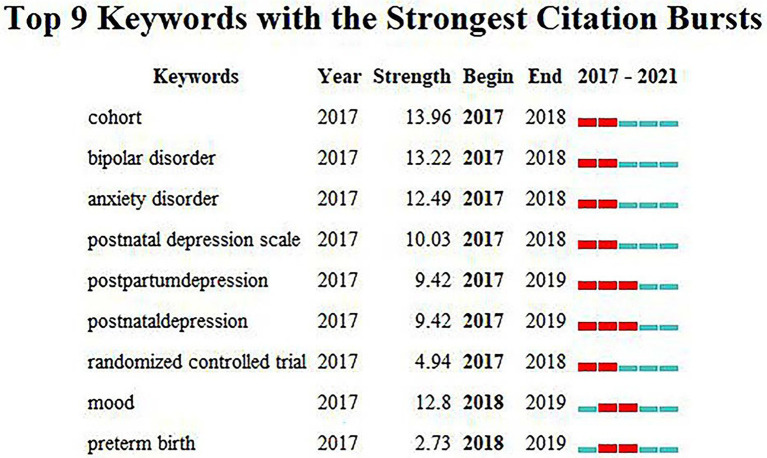
Top 9 Keywords with the Strongest Citation Bursts.

Nine burst keywords were obtained, namely, cohort, bipolar disorder, anxiety disorder, etc., as shown in Figure 9. Keyword burst analysis shows the results from two aspects of burst strength and time. Keywords with high burst strength in a given period of time received great attention in that time interval, which to some extent represents the research frontier in the corresponding time interval (Lin et al., 2020). The burst keywords that appeared in this field can be roughly divided into three time periods. The burst words that appeared from 2017 to 2018 were cohort, bipolar disorder, anxiety disorder, postnatal depression scale and randomized controlled trial. The burst keywords that appeared from 2017 to 2019 were postpartum depression and postnatal depression. The burst keywords that appeared from 2018 to 2019 were mood and preterm birth. According to the analysis results, no burst keywords appeared in 2019–2021, indicating that research in this field is in the stage of continuous exploration and problem discovery.

## Discussion

In this study, CiteSpace software was used to analyze the literature on PPD-related research in the past 5 years and to explore the dynamic evolution and future development trend in this field. From 2017 to 2021, the annual number of publications in this field increased year by year, indicating that research focused in this field was increasing. From the point of view of the source country, research results from the United States, Canada and England were prominent and influential. Most of the PPD research institutions were internationally renowned universities, with the highest number of papers published by the University of Toronto in Canada. Harvard Medical School was the institution with the highest centrality. Among the productive authors, Cindylee Dennis from the University of Toronto, Canada ranked first with 34 articles. The main research directions of Professor Dennis and her team were risk factors for postpartum depression and the relationship between prenatal anxiety and postpartum depression (Dennis et al. 2017b; Shorey et al., 2017; Grigoriadis et al., 2019). It was found that the academic exchanges between institutions and authors’ teams still need to be strengthened, and international cooperation needs to be further expanded.

According to the results of keyword co-occurrence analysis, the research hotspots in this field mainly focused on keywords such as pregnancy, depression, prevalence, symptom, and risk factor. Studies have shown that PPD may adversely affect mothers, infants and families in the short or long term and pose serious public health problems (Julian et al., 2021). Approximately 13–19% of women in high-income countries have PPD after delivery (Lewis et al., 2021). The incidence of depression during pregnancy was 12%, indicating that PPD may start during pregnancy in some cases, and perinatal depression was common (Cuijpers and Karyotaki, 2021). If perinatal depression is not screened and treated in a timely manner, the degree of depression after childbirth may be higher as well as the risk of maternal suicide (Putnam et al., 2017).Counseling interventions, such as cognitive behavioral therapy and interpersonal therapy, have been found to be effective in preventing perinatal depression (US Preventive Services Task Force et al., 2019). Koutra et al. (2018) believed that pregnancy, perinatal and postpartum complications such as gestational hypertension, preeclampsia, sleep disorders, and breastfeeding difficulties would adversely affect the mood of early postpartum women, leading to postpartum depression. In addition to the impact of pregnancy, perinatal and postpartum complications on women’s mental health, Dencker et al. (2019) believed that women’s fear of childbirth during pregnancy would lead to anxiety and depression before or after childbirth. Creating a female-centered childbirth environment where women feel free and safe can reduce the negative psychological states that can arise from a negative or traumatic childbirth.

It is very important to understand the risk factors of PPD and timely screen and treat women with postpartum depression (Peng et al., 2021). Research shows that the risk factors of postpartum depression mainly include stress and anxiety, lack of support, pregnancy related complications, unexpected pregnancy, COVID-19 pandemic, poor economic situation, low education level, etc. (Alshikh Ahmad et al., 2021; Chen et al., 2022). Zejnullahu et al. (2021) conducted a prospective observational cohort study on 247 women who gave birth. Using bivariate and multivariable logistic regression analysis, they determined four predictive variables of PPD, including pregnancy complications, fear of delivery, prenatal depression or anxiety, and poor marital relationship (95% CI, *p* < 0.05). They also found that there was no statistically significant correlation between PPD and maternal age, education level, employment, family type, smoking, previous abortion, parity, etc. Røysted-Solås et al. (2022) reported that mothers who had a history of mental illness and neonatal death had the highest risk of PPD. During the prevalence of COVID-19, Shuman et al. (2022) investigated 670 postnatal patients in the United States and found that compared with mothers who breast fed or bottle fed their own breast milk, the probability of positive PPD screening for mothers who fed infant formula was 92% higher, and the probability of positive screening for severe depression was 73% higher. Different social and cultural factors may also affect the risk factors of PPD. In the future, large sample and high-quality research can be conducted to explore the risk factors of PPD. There are many risk factors of PPD, so it is very important to screen PPD early, eliminate risk factors and treat PPD in time (Hanach et al., 2022).

By analyzing the co-citation clusters and timeline knowledge map, we found that the COVID-19 epidemic is a recent research hotspot. An article published in the *Lancet* highlighted the need to study how to mitigate the impact of the COVID-19 epidemic on people’s mental health, particularly for vulnerable groups (Holmes et al., 2020). Zanardo et al. (2020) found that the Edinburgh Postnatal Depression Scale (EPDS) scores of parturient women who delivered during COVID-19 epidemic isolation in northeastern Italy was significantly higher than that of the control group (*p* = 0.001). Concerns about infection by the novel coronavirus and isolation measures taken during the epidemic have adverse effects on maternal thoughts and emotions and worsen depressive symptoms. Ostacoli et al. (2020) found that women who gave birth during the COVID-19 epidemic had a higher probability of PPD. Scholars believe that early detection of psychological problems during pregnancy is conducive to providing targeted prevention and therapeutic psychological intervention. Lebel et al. (2020) found that during the COVID-19 epidemic, the symptoms of anxiety and depression in pregnant women increased significantly, which can have long-term adverse effects on their children. Moreover, they believed that the adverse effects can be alleviated by increasing social support for pregnant women and strengthening their physical exercise.

Based on comprehensive analysis, the risk factors of PPD such as prenatal depression or anxiety, fear of delivery, etc., and the impact of COVID-19 pandemic on PPD are the research focuses of PPD in recent 5 years. The limitation of this study is that it only used CiteSpace to visually analyze the English literature related to this field in the Web of Science Core Collection, and the included literature was not comprehensive enough. Therefore, the results obtained in this study do not represent global research changes in this field. Thus, the results obtained from this study are biased to a certain extent.

## Author contributions

RW and YS: conceptualization, methodology, formal analysis, investigation, resources, writing review, and editing. LW: software and data curation. RW: validation and writing original draft preparation. YS: visualization, supervision, and project administration. All authors contributed to the article and approved the submitted version.

## Funding

This work was supported by the Development Course of the Professional Concept of Rehabilitation Therapy in China in the 20th Century (2021KJSKFKT-A03).

## Conflict of interest

The authors declare that the research was conducted in the absence of any commercial or financial relationships that could be construed as a potential conflict of interest.

## Publisher’s note

All claims expressed in this article are solely those of the authors and do not necessarily represent those of their affiliated organizations, or those of the publisher, the editors and the reviewers. Any product that may be evaluated in this article, or claim that may be made by its manufacturer, is not guaranteed or endorsed by the publisher.
